# Estimation of Quasi-Stiffness and Propulsive Work of the Human Ankle in the Stance Phase of Walking

**DOI:** 10.1371/journal.pone.0059935

**Published:** 2013-03-21

**Authors:** Kamran Shamaei, Gregory S. Sawicki, Aaron M. Dollar

**Affiliations:** 1 School of Engineering and Applied Science, Department of Mechanical Engineering and Materials Science, Yale University, New Haven, Connecticut, United States of America; 2 Joint Department of Biomedical Engineering, North Carolina State University and University of North Carolina at Chapel Hill, Raleigh, North Carolina, United States of America; University of Rochester, United States of America

## Abstract

Characterizing the quasi-stiffness and work of lower extremity joints is critical for evaluating human locomotion and designing assistive devices such as prostheses and orthoses intended to emulate the biological behavior of human legs. This work aims to establish statistical models that allow us to predict the ankle quasi-stiffness and net mechanical work for adults walking on level ground. During the stance phase of walking, the ankle joint propels the body through three distinctive phases of nearly constant stiffness known as the quasi-stiffness of each phase. Using a generic equation for the ankle moment obtained through an inverse dynamics analysis, we identify key independent parameters needed to predict ankle quasi-stiffness and propulsive work and also the functional form of each correlation. These parameters include gait speed, ankle excursion, and subject height and weight. Based on the identified form of the correlation and key variables, we applied linear regression on experimental walking data for *216* gait trials across *26* subjects (speeds from *0.75–2.63 m/s*) to obtain statistical models of varying complexity. The most general forms of the statistical models include all the key parameters and have an R^2^ of *75%* to *81%* in the prediction of the ankle quasi-stiffnesses and propulsive work. The most specific models include only subject height and weight and could predict the ankle quasi-stiffnesses and work for optimal walking speed with average error of *13%* to *30%*. We discuss how these models provide a useful framework and foundation for designing subject- and gait-specific prosthetic and exoskeletal devices designed to emulate biological ankle function during level ground walking.

## Introduction

Several engineering fields desire a better understanding of human locomotion biomechanics including anthropomorphic bipedal robots [1], [2], lower-limb wearable exoskeletons [3]–[10], and biologically-inspired prosthetic limbs [11]–[14]. Emulation of human locomotion in these artificial systems would ideally be built upon theoretical or empirical models that can accurately characterize the behavior of lower extremity joints during gait [15]–[17]. Theoretical and empirical models of varying complexity for the whole leg and for the compliant components have been investigated by other researchers and can be used in these systems to help generate human-like locomotion [1], [17]–[25]. At a joint level, researchers typically characterize the kinetic and kinematic behavior of the joints using data experimentally captured in a gait laboratory [26]–[28]. Others have studied the passive and active stiffness of the joints using system identification techniques that employ statistical analyses and experimental data [29]–[31]. A common finding from all of these approaches is that compliance, both at the whole-limb and individual joint level, plays a central role in shaping human motion.

The compliance of lower extremity joints during locomotion can be investigated by the concept of quasi-stiffness or “dynamic stiffness” [32]–[42]. The term quasi-stiffness is usually reserved for lower extremity joints (e.g. ankle, knee, and hip) and can be distinguished from the passive and active stiffness of a joint typically used to describe the ‘local’ tangent to the moment-angle curve exhibited for a given joint at a specific angle and for a certain level of muscle activation as described in the literature [29], [30]. The quasi-stiffness of a joint is defined more globally, as the slope of the best linear fit on the moment-angle graph of a joint over a whole stride or specific phase of a stride [32]–[40]. The ankle joint is primarily involved in the propulsion of the body during the stance phase. The concept of quasi-stiffness can be applied to characterize the ankle behavior in the propulsion period of stance, where the ankle demonstrates two distinctive stages: a rising extensor moment stage that stores energy and a falling extensor moment stage that returns energy [34]. Our preliminary investigation of the ankle quasi-stiffness revealed linear behavior in the energy return stage of stance that changes with gait speed, ground slope, and load carriage [37], which is in agreement with the results of [13]. Here, we divide the storage stage into two subsequent phases, which are divided at the time halfway through this stage: a. dorsi-flexion and b. dual-flexion (similar to phases shown in [34]). In this work, we show that the ankle exhibits nearly linear behavior in dorsi-flexion and dual-flexion.

Many designers of orthoses and prostheses have sized their devices based on the average kinetic and kinematic data of humans [10], [44]. In contrast, the overall goal of this study was to establish a series of statistical models, aimed to inform the stiffness design or control of ankle-foot orthoses and prostheses, to characterize the linear behavior of the ankle during propulsion for adult humans as a function of body size (height and weight) across a range of walking speeds, *without requiring the gait of a specific subject to be analyzed*. These models of ankle joint stiffness during walking promise to aid in diagnosis of musculoskeletal dysfunction and the development of biologically-inspired assistive devices (orthoses and prostheses) to improve mobility [45]. For the latter applications, the level of compliance of the ankle joint will often need to be chosen in advance to provide versatile user-adaptability (e.g. in [46], [47]) or in a real-time adaptive-controller to provide gait adaptability (e.g. in [12]). For these applications, generalized biomechanical models that can characterize subject-specific and gait-specific variability of the behavior of lower extremity joints will be critical for sizing devices (e.g. choosing actuator power and spring stiffness) to individual users and gaits.

We begin this paper with a description of the ankle behavior and parameters of interest during walking, as well as data collection methods used in the study. We extract a generic equation for the ankle moment through an inverse dynamics analysis. Based on this equation, we identify a subset of independent factors that can describe the quasi-stiffness and mechanical work of the ankle during gait. Next, we employ a considerably comprehensive experimental data set (216 gait trials across 26 subjects) to fit coefficients to these terms and establish statistical models for the ankle quasi-stiffness and work as functions of walking speed (*V*) and ankle excursion, as well as the individual’s height (*H*) and weight (*W*). There are many applications where a priori knowledge of the ankle excursion is not available and only one stiffness is required, such as “sizing” compliant ankle prostheses or orthoses that are versatile enough to perform around the optimal gait speed, without needing time-consuming ‘on-board’ measurements. For these cases, we try to establish simpler models that only include height and weight, at the expense of reduced accuracy.

## Methods

### Ankle Phases of Motion in a Gait Cycle

**Figure 1:**
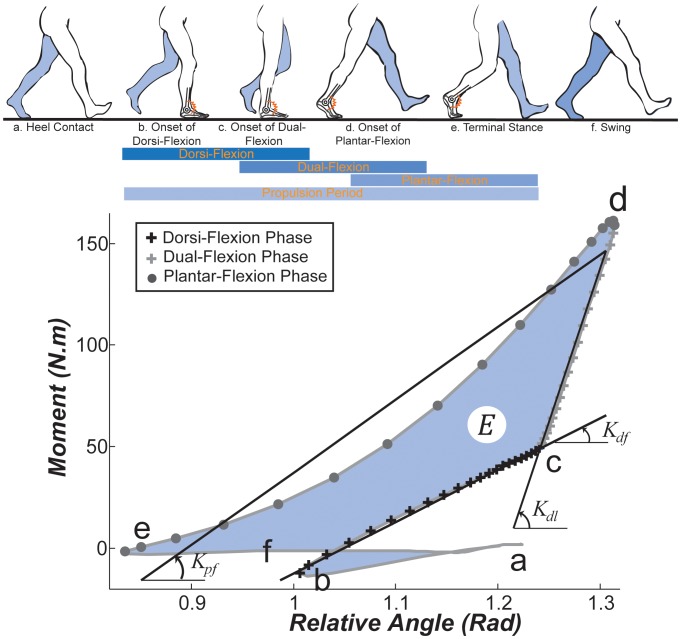
Ankle moment vs. relative angle curve for a representative subject walking at 1.75 *m/s*. Letters a-f on the graph correspond to the poses schematically shown during a typical walking cycle (top, schematic timing is adapted from [69]). Quasi-stiffness is calculated based on the slope of the best-line fit to the moment-angle curve of b-c for the dorsi-flexion (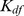), c-d for the dual-flexion (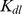), and d-e for the plantar-flexion (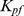) phases of the progression period (b-e). The area enclosed by the graph represents the propulsion work of the ankle (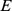). The joint excursion in each phase is the difference between the ankle relative angle at the onset and end of that phase (i.e. ,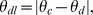 and 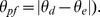

The gait cycle can be divided into the stance and swing phases as schematically shown in Fig. 1, top. The ankle exhibits an initial plantar-flexion motion within the first ∼10% of the gait (Fig. 1, a-b) until the foot sole lays on the ground [48]. Within the rest of the stance phase, the ankle is primarily involved in the progression of the body [49]. The ankle undergoes three sub-phases during the progression period including dorsi-flexion (Fig. 1, b-c), dual-flexion(Fig. 1, c-d), and plantar-flexion(Fig. 1, d-e) phases [34]. Next, the toe leaves the ground and the ankle experiences a relatively silent swing phase (Fig. 1, e-a). The dual-flexion phase ends at ∼50% of the gait cycle [34]. The dorsi-flexion and dual-flexion phases separate at ∼30% of the stride when the ground reaction force shows a local minimum in the vertical and zero in the horizontal directions [26]. We adopt the term dual-flexion because in that phase the ankle demonstrates dorsi-flexion motion at slow and plantar-flexion motion at fast gait speeds. This study centers on the progression period (Fig. 1, b-e).

### Terminology: Quasi-Stiffness, Propulsive Work and Angular Excursion of the Ankle

This work characterizes the ankle quasi-stiffnesses (*N.m/rad*) during the dorsi-, dual-, and plantar-flexion phases, as well as the propulsive work (*J*) performed in the progression period. We define the quasi-stiffness of the dorsi-flexion phase (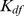), dual-flexion (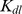), and plantar-flexion (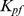) as the slopes of the lines fit to the moment-angle graph of the ankle in the corresponding phase (see Fig. 1, bottom). We obtain the magnitude of excursion of the ankle in the dorsi-flexion (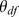), dual-flexion (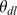), and plantar-flexion (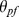) phases by subtracting the initial angle from the final angle in that particular phase (e.g. 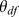 is obtained by subtracting the ankle angle at instant *b* from the ankle angle at instant *c*, which implies that it is independent of the actual angle of the joint at *b* and *c*). The area enclosed by the moment-angle graph equals the propulsive mechanical work of the ankle in the progression phase (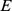), and approximately equals the ankle work over the whole gait cycle because the ankle is nearly silent during the rest of the stride.

### Identifying the Model Parameters and Form of Fits

The generic analytical equation (1) for the ankle moment was obtained through a general inverse dynamics analysis (as documented in the Appendix, Fig. S1 and Table S1). To identify the key parameters of the models and their functional forms, we simplified the generic equation for the ankle moment to extract the ankle moment in the sagittal plane *only* (*X-Y* of Fig. S1) for the instants of maximum moment in the dorsi-flexion and dual-flexion phases (Fig. 1, point c and d). Then, we extracted the forms of models and potential parameters by investigating the terms of the simplified equation for the ankle moment and correlating them with body and gait parameters. Here, the weight (*W*) and height (*H*) are considered as the body parameters; whereas, the walking speed (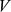), and magnitude of ankle excursion in dorsi-flexion (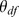), dual-flexion (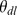), and plantar-flexion (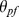) are considered as the gait parameters.

The moment of the ankle is given by the following analytical equation obtained through the inverse dynamics analysis outlined in the Appendix:
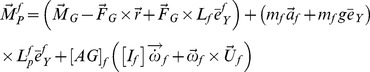
(1)


Table S1 lists definitions for the parameter of equation (1) and the equations that follow.

The ground reaction force (GRF) exhibits a local minimum around the instant of transfer from dorsi- to dual-flexion and a local maximum around the instant of transfer from dual-flexion to plantar-flexion phase in normal walking on a level ground [26], [50]. We neglect the ground reaction moment (GRM) because it is substantially smaller than the ankle moment (i.e. 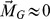). Since the support foot is instantaneously nearly stationary and the angular momentum of the foot segment is substantially smaller than the rest of the body at these two instants of phase transfer (Fig. 1, points c and d), we also neglect the effect of foot angular velocity (i.e. 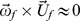). Next, since the foot is dramatically loaded at these instants to propel the rest of the body, we neglect the effects of linear and angular acceleration. Moreover, we neglect the effect of the weight of the foot as it is small compared to that of the rest of the body (i.e. 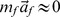 and 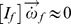). Applying all these assumptions in equation (1) provides us with the following reduced expression for the ankle moment at phase transitions (points c and d):
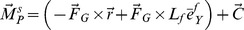
(2)


To be more accurate, we introduce 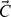 to reflect the effect of the neglected terms. As indicated in Table S1, 
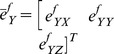
 and 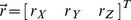 are vectors. Therefore, the moment of the ankle in the sagittal plane is the *Z*-component of equation (1) as follows:
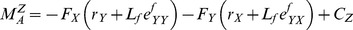
(3)where, 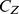 is the 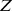-component of 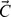 and 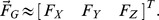


#### Dorsi-Flexion phase

At the instant of transfer from dorsi-flexion to dual-flexion phase, 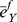 is assumed to be constant because the foot is instantaneously stationary. Previous research shows that the center of pressure (COP) tends to approximately lay at the middle of the foot sole [51] at this instant. Therefore, 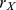 would be correlated with 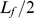, and hence with 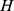, making 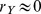. Based on anthropometric relationships, 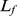 scales in proportion to 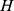 [52]. Thus:
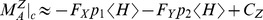
(4)where, 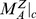 is the moment of ankle in the sagittal plane at the instant of transfer from dorsi- to dual-flexion (Fig. 1, point *c* which happens at ∼30% of the gait cycle). In this text, 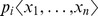 denotes an arbitrary first-order polynomial of 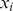
’s. Previous research indicates that the extrema of the normalized GRF (particularly the value of local minimum of vertical component in the dorsi-flexion phase here denoted by 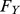) is correlated with the gait speed and the horizontal component of the ground reaction force is nearly negligible [26], [50]. In other words, at the instant of transfer from dorsi-flexion to dual-flexion phase we have:



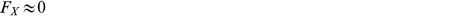
(5–a)

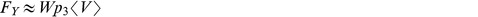
(5–b)


Applying equations (5-a and b) in equation (4) concludes:
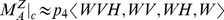
(6)


Previous studies suggest that the ankle behaves nearly linearly in the dorsi-flexion phase of the gait [34], [36]. We observed similar behavior for the experimental subjects of this study (Table 1), as we found an average 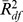 of *96%*, for a linear fit to the moment-angle curve during dorsi-flexion phase. This implies:
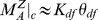
(7)


**Table 1:** Details on Subjects and Experimental Trials used for Regression Fits.

Subject	Gender	#Trial	*W*	*H*	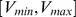	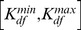	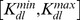	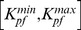	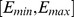	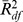	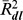	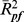
1‡	M	4	92.3	1.86	[0.75,2]	[248,440]	[−4277,3837]	[233,314]	[3.1,38.1]	92	96	92
2‡	M	4	68.4	1.70	[0.75,2]	[138,184]	[389,6944]	[238,300]	[0.9,24.1]	99	85	96
3‡	M	4	65.6	1.65	[0.75,2]	[202,323]	[−864,9858]	[203,286]	[−3.7,22.6]	93	75	91
4‡	M	4	94.0	1.86	[0.75,2]	[248,353]	[−12362,1525]	[348,404]	[−9.1,24]	99	86	94
5‡	M	4	68.1	1.72	[0.75,2]	[179,220]	[394,2987]	[236,255]	[0.2,23.9]	99	91	93
6‡	F	4	57.7	1.43	[0.75,2]	[107,180]	[271,5172]	[165,237]	[−2.0,17.4]	98	86	94
7‡	F	4	63.1	1.45	[0.75,2]	[81,192]	[−862,1141]	[94,188]	[8.7,28.0]	98	84	95
8‡	F	4	65.7	1.75	[0.75,2]	[156,175]	[−1849,14888]	[174,216]	[4.5,26.5]	98	74	93
9‡	F	4	75.9	1.80	[0.75,2]	[245,387]	[−7841,579]	[192,249]	[4.3,32.1]	98	89	96
10†	M	20	85.7	1.74	[1.26,2.43]	[266,672]	[496,2160]	[183,273]	[7.3,43.6]	99	94	92
11†	M	20	79.2	1.82	[1.38,2.25]	[22,406]	[−62897,33953]	[133,265]	[14.5,59.8]	91	47	94
12†	M	20	62.1	1.64	[1.04,2.29]	[68,214]	[−13140,4939]	[152,222]	[1.7,22.5]	98	83	91
13†	M	20	62.0	1.62	[1.01,2.44]	[141,311]	[−25694,43369]	[132,225]	[−4.5,20.1]	98	83	92
14†	M	20	75.1	1.77	[1.30,2.63]	[205,402]	[−6162,18607]	[203,288]	[22.4,38.3]	95	77	95
15•	F	5	58.0	1.60	[1.00,1.25]	[130,235]	[−1416,4355]	[129,154]	[10.9,22.2]	94	37	93
16•	F	6	56.0	1.60	[1.18,1.26]	[140,244]	[396,2870]	[123,159]	[11,19.3]	97	47	97
17•	F	9	48.0	1.58	[0.96,1.08]	[162,263]	[−629,406]	[138,233]	[9.9,18.7]	99	72	98
18•	F	7	46.0	1.60	[1.08,1.19]	[116,183]	[393,4321]	[79,113]	[0.3,7.5]	95	69	81
19•	F	4	53.0	1.61	[1.12,1.28]	[51,299]	[−1170,402]	[129,194]	[−0.2,6.9]	94	74	85
20•	F	5	53.0	1.67	[1.3,1.34]	[113,209]	[434,778]	[135,165]	[5.2,7.1]	95	99	87
21•	M	7	90.0	1.80	[1.24,1.31]	[222,351]	[−856,5780]	[230,281]	[17.7,34.5]	95	45	96
22•	M	9	55.0	1.73	[1.18,1.26]	[138,191]	[425,2352]	[135,194]	[9.9,20.2]	98	80	98
23•	M	5	77.0	1.80	[1.36,1.42]	[226,356]	[−906,1805]	[136,183]	[13.3,27.6]	95	57	92
24•	M	4	75.0	1.87	[1.39,1.48]	[180,277]	[595,1224]	[194,214]	[12.3,14.9]	92	82	89
25•	M	6	71.0	1.72	[1.27,1.35]	[202,482]	[−7211,10258]	[221,308]	[13.8,19.4]	96	32	94
26•	M	13	72.0	1.81	[1.13,1.27]	[217,316]	[−15853,3456]	[167,262]	[6.3,22.3]	96	67	91
	**Mean**		**69.1**	**1.71**	**1.51**	**246**	**992**	**202**	**17.6**	**96**	**73**	**93**
	**SD**		**12.4**	**0.10**	**0.41**	**98**	**7061**	**53**	**10.8**	**7**	**32**	**5**

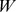
: Body weight (kg), and 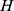: Body height (m).

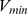
 and 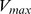: Minimum and maximum gait speed (m/s).

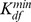
 and 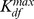: Minimum and maximum quasi-stiffness in dorsi-flexion phase (Nm/rad).

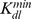
 and 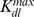: Minimum and maximum quasi-stiffness in dual flexion phase (Nm/rad).

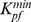
 and 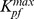: Minimum and maximum quasi-stiffness in plantar-flexion phase (Nm/rad).

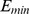
 and 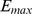: Minimum and maximum propulsion energy (J).

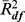
, 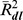, and 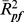: Average 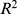 of the linear fit on moment-angle curve in dorsi-flexion, dual-flexion, and plantar-flexion phases.

‡ Data collected at Human PoWeR Lab, NC State University [28].

† Data collected at Biomechanics Lab, East Carolina University [43].

•Data collected at Laboratory of Biomedical Technologies at Politecnico Di Milano.

Combining (6) and (7) constitutes the following analytical form for the quasi-stiffness of the ankle in the dorsi-flexion phase:
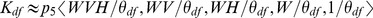
(8)which suggests that the quasi-stiffness of the ankle in the dorsi-flexion phase could be characterized by a first order polynomial of 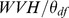, 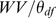, 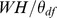, 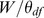, and 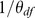.

#### Dual-Flexion phase

At the instant of transfer from dual-flexion to plantar-flexion, the heel is off and the toe is on the ground. Thus, 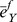 makes an angle (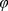) with the *X*-axis. Also, it has been shown that in this phase, the center of pressure (COP) approximately lays at the rear of the toe and close to the heads of the metatarsi [48], [51]. Therefore, 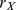 would be correlated with the length of toe, and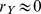. Anthropometric relationships imply that the toe length is proportional to 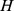 [52]. Therefore:
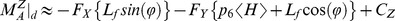
(9)where, 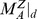 is the moment of ankle in the sagittal plane at the instant of transfer between dual- and plantar-flexion. Previous research shows that the extrema of the normalized GRF (especially the value of maxima of vertical and horizontal components during the push-off phase, here denoted by 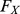 and 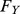) are correlated with the gait speed for normal walking on level ground [50]. In other words, at the transfer instant between the dorsi- and dual-flexion phases we have:



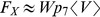
(10–a)

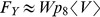
(10–b)


Researchers have also investigated the variability of foot kinematics under three gait speeds [53]. They have shown that the maximum value of hallux dorsi-flexion significantly increases as the gait speed increases. Considering these findings, we assume that the foot orientation (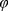, as shown in Fig. S1) could be approximated by a linear function of the gait speed (i.e. 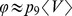). Applying this approximation and equations (5-a and b) in equation (4) results in:
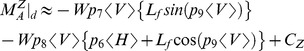
(11)


Approximating the trigonometric functions by the first two terms of their Taylor series yields:
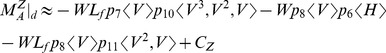
(12)which can be further reorganized as:
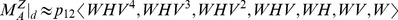
(13)


Previous research suggests that the ankle behaves nearly linearly in dorsi- and dual-flexion phases of the gait [34]. For the dual-flexion phase, however, we observed 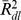 of less than *50%* in *47* gait cycles out of *216,* mostly near a singular gait speed where the ankle exhibits a transient behavior from dorsi-flexion to plantar-flexion behavior. At speeds above and below this singular speed, we observed linear behavior. Therefore, for all the gait trails except at the singular speed we have:
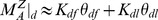
(14)


Using equations (8), (13), and (14), we obtained the following for the quasi-stiffness of the ankle in the dual-flexion phase:
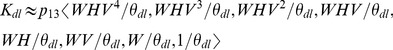
(15)which suggests that the quasi-stiffness of the ankle in the dual-flexion phase of the gait could be captured by a first order polynomial of 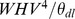, 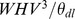, 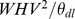, 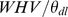, 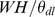, 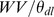, 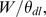 and 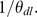


#### Plantar-Flexion phase

We use a similar approach for the plantar flexion phase. Previous research suggests the ankle behaves nearly linearly in the plantar-flexion phase of stance [34], [36], [37]. Similar behavior is observed in the current study where the subjects in average exhibited 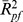 of *93%*. Therefore:
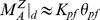
(16)


Identical left hand-sides of equations (13) and (16) suggest the following analytical form for the quasi-stiffness of the ankle in the plantar-flexion phase of stance:
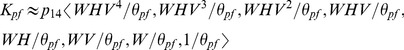
(17)which suggests that we can model the quasi-stiffness of the ankle in the plantar-flexion phase by a first order polynomial of 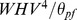, 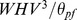, 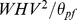, 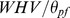, 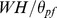, 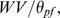 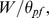 and 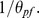


#### Propulsive work

The area enclosed by the moment-angle graph equals the propulsive mechanical work of the ankle in the gait cycle and particularly in the stance phase, because the ankle is nearly silent in the swing phase of the gait. The linear behavior of the ankle in the dorsi-, dual-, and plantar-flexion phases implies that we can estimate the mechanical work by the area enclosed by the regression lines depicted in Fig. 1. Therefore, the propulsive work of the ankle could be estimated by:
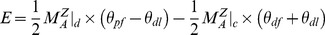
(18)


Combining (6) and (13) into (18) constitutes the following form for the propulsive work of the ankle:
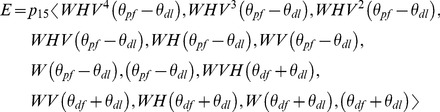
(19)


### Experimental Protocol, Data Extraction and Statistical Analysis

The ankle angle and moment data for 26 human subjects (the numbers of trials are listed in Table 1) were provided to us by other researchers from previous studies from three labs:

1. Nine subjects (subjects 1 to 9 in Table 1) at Human PoWeR Lab, NC State University walking on a treadmill, as detailed in [28];
2. Five subjects (subjects 10 to 14 in Table 1) at Biomechanics Lab, East Carolina University walking on level ground. The general procedures used to obtain the ground reaction force, sagittal plane knee joint angular position and torque are described in detail in [43]. We detail here the specific procedures relevant to the purpose of this study. All participants read and signed an informed consent form approved by the University Institutional Review Board at East Carolina University. Using a 15 m walkway, force platform (AMTI, Watertown, Ma) and eight camera motion capture system (Qualisys, Gothenberg, Sweden), three dimensional ground reaction force and linear position data describing the right lower extremity and pelvis were obtained from each participant during 20 walking trials of different velocities ranging from 1.01 to 2.63 *ms^−1^*. Each participant was initially tested at a self-selected, moderate walking speed the mean of which was 1.63±0.03 *ms^−1^*. Subsequently, the 19 remaining trials per participant were collected in an approximately random order of walking velocities. Participants were instructed to walk at various speeds with instructions such as, “walk at a moderately fast pace,” “walk at a very slow pace,” and “walk at your fastest pace.” The mean walking velocity for all trials was 1.77±0.36* ms^−1^*. All participants had similar minimum and maximum walking velocities and therefore similar ranges of walking velocities. Additionally, the 20 walking velocities for each participant were moderately evenly distributed through the range of velocities from slowest to fastest velocities. Qualisys Track Manager and Visual 3D software (C-Motion, Gaithersburg, Md) were used to calculate the knee joint angular position and torque through the stance phase of walking in each trial from the linear position and ground reaction force data. The subject consents, collection protocols and data analysis for subject groups 1 and 2 are detailed in [28], [43], respectively.
3. Twelve subjects (subjects 15 to 26) at Laboratory of Biomedical Technologies at Politecnico Di Milano walking on level ground. For subject group 3, kinematic data were collected by using a motion analyzer (ELITe System, BTS, Italy) based on TV-signals processing [54]. Retroreflective markers were positioned on the body according to a predefined protocol [55], [56]. Eight TV-cameras were located in the laboratory as to detect a calibrated volume 3 m long, 2 m high, 1.5 m wide. Accuracy of the 3D coordinates was approximately 1 mm in the calibrated volume; frequency of acquisition was 50 *Hz*. Kinetic data were obtained by measuring ground reaction forces and moments through a dynamometric force platform (Kistler 9281B, Winterthur, Switzerland). Data processing to estimate joint centers and to compute joint moments, based on an inverse dynamics approach, has been described by [57], and was validated, more recently, in a comparative study performed by [58].

Subjects 1 to 14 walked with a wide range of gait speeds (*0.75–2.63 m/s*); whereas, subjects 15 to 26 only walked at their preferred speed. This study only includes unimpaired male and female adults with a reasonably wide range of masses (*46–94.0 kg*) and heights (*1.43–1.87 m*). We analyzed the moment-angle graphs for each subject (similar to Fig. 1-bottom). To distinguish the phases, the onset of the dorsi-flexion phase was identified as the point of local minimum angle after the heel contacts the ground (point *b*) and the end of dual-flexion phase as the point of maximum moment (point *d*). The instant of transfer from dorsi-flexion to dual flexion was chosen as ∼30% of the gait cycle (point c). The end of plantar-flexion phase was chosen as the point of minimum angle in the gait (point *e*). As such, the dorsi-flexion phase is composed of the data points between *b* and *c*; the dual-flexion *c* and *d*, and the plantar-flexion phase *d* and *e*. Assuming accuracy of the measurements, we applied linear fits between the angle and moment data points, using method of least square regression, and extracted the slopes in each phase corresponding to 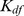, 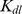, and 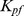 (as described in the previous section).

The previous section outlines several collinear predictors for the models of 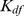, 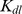, 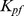, and 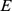. To establish predictive models that are composed of many collinear predictors, we first cross-validated the models structures by removing one subject at a time (stratified cross-validation) and applying Partial Least Square (PLS) analysis to evaluate the predictive ability of the chosen parameters and to find the optimal number of components that could best describe the response variables (i.e. quasi-stiffnesses and work) [64]–[66]. Next, we applied the linear regressions between the values of 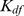, 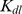, 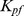, and 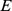 and the parameters that the previous section suggested. We chose least square regression because we assumed the predictor parameters are known (i.e. accurately measured). We started with a linear regression that included all the key parameters. Stepwise, non-significant terms (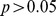) of the regressed polynomials were iteratively removed until we reached a polynomial that only included terms with significant coefficients. We termed these polynomials as the *general-form models*.

### Stature-Based Models

Researchers have shown that people with different body sizes prefer to walk at the nondimensional optimal gait speed of 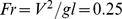, where *l* is the leg length and *g* is the gravitational acceleration [59]–[63]. We used this relationship to relate the optimal walking speed to the subject’s stature (*H* and *W*). Assuming an anthropometric relationship of 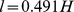 [52], the optimal or “preferred” gait speed is approximated by:
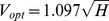
(20)


The general models also include the ankle excursion, which is not usually known a priori for a given individual. Therefore, we intend to exclude it from the general-form models. However, we did not observe any inter-subject relationship between the ankle excursion and the body parameters. Among the quasi-stiffnesses, we observed that 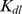 is highly sensitive to 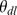 when 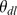 is small and also when gait speed is substantially lower than the preferred gait speed. However, the ankle quasi-stiffnesses demonstrated less dependence on the amount of excursion at the preferred gait speed. Therefore, we applied one possible method to exclude the ankle excursion from the general models and used the average values of 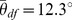, 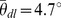, and 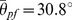 observed at the preferred gait speeds.

Using equation (20) and plugging in the average values for the ankle excursion in the general-form models provided us with a series of *stature-based models* that predict the quasi-stiffnesses and propulsive work of the ankle at the preferred gait speed only as a function of 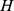 and 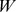.

## Results

We observed relatively linear behavior for nearly all subjects and gait speeds in dorsi-flexion and plantar-flexion phases of stance. During the dual-flexion phase, the ankle behaved linearly at most gait speeds; except we found a singular gait speed for subjects 1 to 14 around which the ankle deviated from a linear behavior. For these 14 subjects, the singular speed was higher than the preferred speed. We only observed that singular speed in only a few gait cycles for subjects 15 to 26 who only walked at their preferred speed. This suggests that the singular gait speed is higher than the preferred gait speed. Linear fits (similar to that shown in Fig. 1-bottom) demonstrated an average 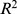 of 96% in the dorsi-flexion, 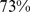 in dual-flexion (including the singular speeds), and 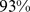 in plantar-flexion phases (Table 1). For each subject, the minima and maxima of the ankle joint quasi-stiffnesses and propulsive work as well as the average values of 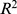 are also reported for different phases in Table 1. The average values of 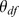, 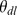, and 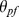 were calculated as 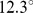, 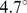, and 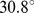.

Table 2 shows that the cross-validation analyses suggest 3,4,3, and 9 components of equations 8, 15, 17, and 19 for the general-form models of 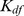, 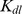, 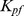, and 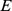. Table 2 also includes the values of 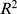 and predicted 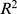 for the cross-validation analysis. Next, we applied Least Square Regression to obtain the general-form models as listed in Table 2. We started the regression with all the components that the inverse dynamics analysis suggested for each parameter as outlined in Methods Section and removed the components that were not statistically significant. Table 2 shows the general form models for estimation of 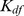, 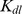, 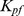, and 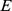. From the 216 gait trials, only 6, 2, 4, and 5 data points demonstrated outlier behavior in the regression analysis for 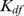, 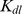, 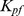, and 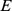, respectively. Table 2 also indicates the average error values of 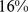, 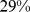, 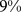, and 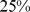 for each model over the entire data sets excluding the outliers. The values of 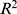 and 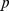 were (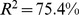, 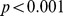) for 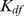, (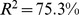, 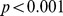) for 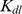, (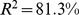, 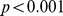) for 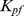, and (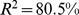, 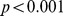) for 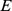 as reported in Table 2. The regression analyses showed 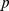-values of 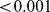 for all of the coefficients of the polynomials, with the exception of 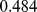 for the intercept in equation (8), which implies that the intercept is negligible, and 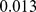 for the intercept in equation (17). We did not observe any notable correlation between the residuals and the order of data collection and magnitude of the quasi-stiffness and work. Except we found slightly greater values for the residuals of the data of subjects 10 to 14 collected at East Carolina University. The residuals of all four fits were also normally distributed.

**Table 2:** General-Form Models to Predict the Quasi-Stiffness and Work of the Ankle Joint for Level Ground Walking.

Phase	Model	Unit	Error	PLS-CV #Comp.	PLS-CV R^2^	PLS-CV Predicted R^2^	Fit Quality
**Dorsi-Flexion**	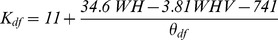	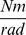	16%	3	75.7%	71.1%	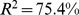 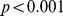
**Dual-Flexion**	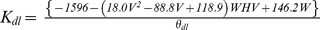	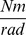	29%	4	71.7%	62.0%	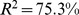 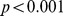
**Plantar-Flexion**	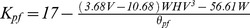	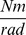	9%	3	81.5%	77.4%	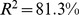 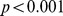
**Stance**	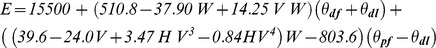	*mJ*	25%	9	78.6%	70.2%	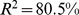 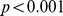

**Figure 2:**
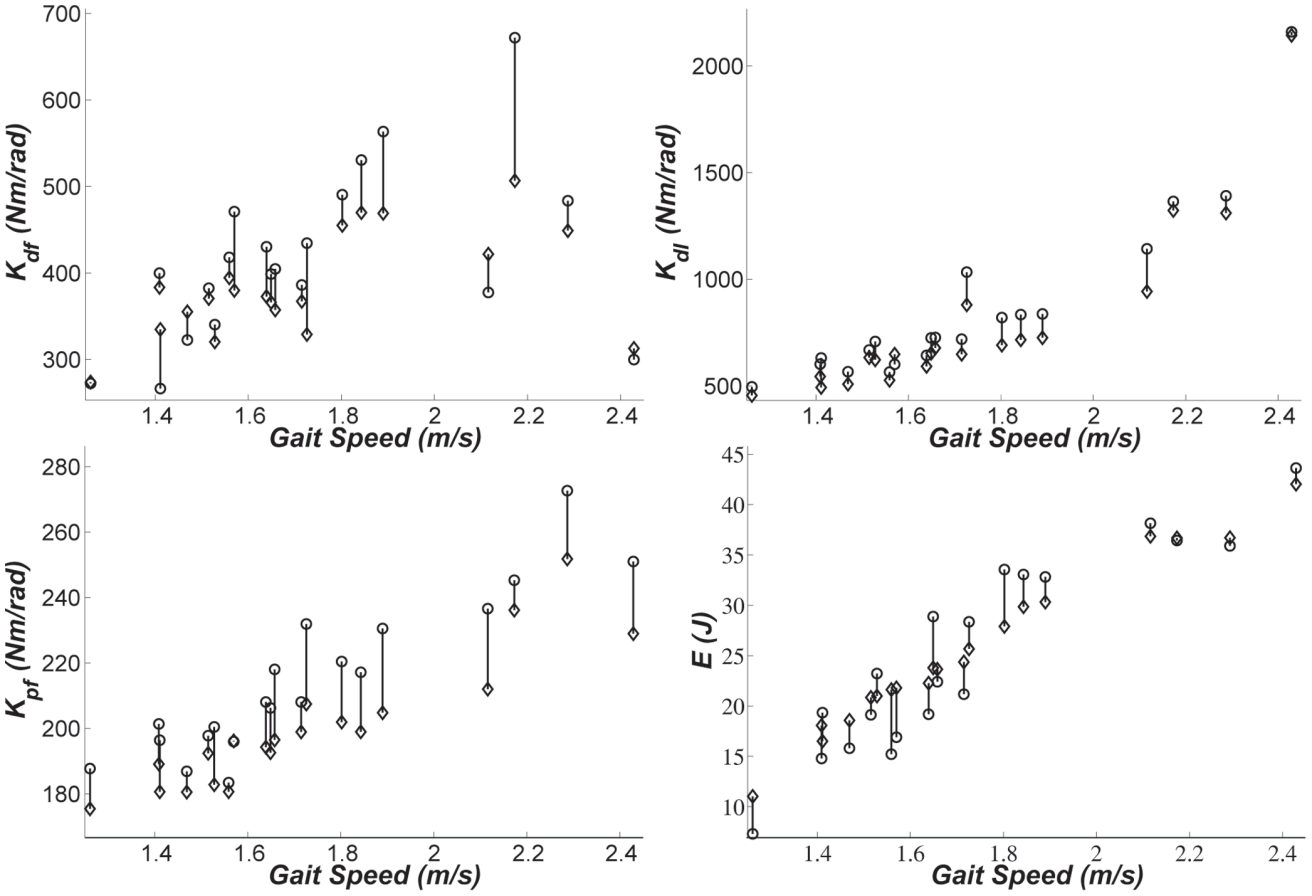
Ankle quasi-stiffnesses (*N.m/rad*) in dorsi-flexion (top-left), dual-flexion (top-right), and plantar-flexion (bottom-left) phases, and propulsive work (*J*) in stance (bottom-tight) plotted against gait speed for subject 10 as an example. The circles indicate the experimental value and the diamonds are the predictions of the general-form models of Table 2.

As an example, we have shown the predictions of the general-form models for subject 10 in Fig. 2. In this plot, both experimental values for the quasi-stiffnesses and propulsive work and the results of the general models are depicted.

**Figure 3:**
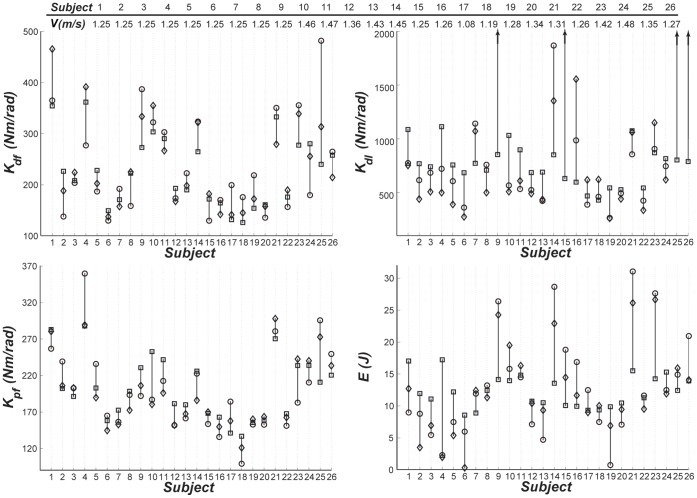
Ankle quasi-stiffnesses (*N.m/rad*) in dorsi-flexion (top-left), dual-flexion (top-right), and plantar-flexion (bottom-left) phases, and propulsive work (*J*) in stance (bottom-tight) plotted for different subjects walking at a speed closest to the preferred gait speed. The experimental values are shown by circles, the predictions of the general-form models by diamonds, and the stature-based models with squares. To avoid suppressing the rest of the data, the arrows are included on the top-right graph to indicate the values that are dramatically higher than the rest of the data.

The stature-based models are reported in Table 3. We cannot report 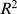 of the stature-based models (23-a to d) because we do not know the “true” optimal gait speed for each subject. To evaluate the accuracy of the stature-based models, we calculated the Froude Number, 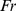 for each gait trial and chose the trial with the speed that is closest to 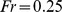 for each subject as illustrated in Fig. 3. Our analysis demonstrates that the stature-based models can predict 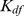, 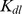, 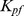, and 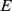 with average errors of 20%, 29%, 13%, and 30% with 2,4,0, and 1 outliers, respectively. This could be compared with the predictions of the general-form models for 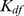, 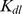, 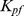, and 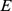 at the optimal gait speed that have 20%, 22%, 9%, and 30% error with 0,1,0, and 1 outliers, respectively.

**Table 3:** Stature-Based Models to Predict the Quasi-Stiffness and Work of the Ankle Joint for Walking at the Optimal Gait Speed on Level Ground.

Phase	Model	Unit	Error	Simplification Method
**Dorsi-Flexion**	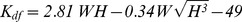	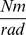	20%	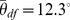 , 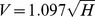
**Dual-Flexion**	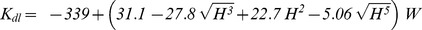	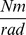	29%	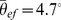 , 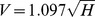
**Plantar-Flexion**	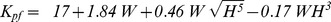	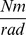	13%	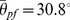 , 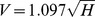
**Gait Cycle**	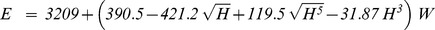	*mJ*	30%	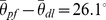 , 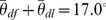 , 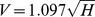

## Discussion

In this paper we have established statistical models that can estimate the quasi-stiffnesses and mechanical work output of the ankle during the stance phase of human walking. To obtain the models, we extracted the generic equation of the ankle moment through an inverse dynamics analysis and simplified it for the stance phase. The simplified equation for the stance phase emphasizes that the quasi-stiffnesses of the ankle are linearly correlated with combinations of both gait and body parameters in the most general form. Using a wide experimental data set and least square linear regression, we constituted expressions that statistically best describe the quasi-stiffnesses of the ankle in the dorsi-flexion, dual-flexion, and plantar-flexion phases, as well as the propulsive work of the ankle in stance. In addition, we developed more simplified and subject-specific (i.e. stature-based) models that are independent of ankle excursion and gait speed. Both of these model frameworks might be used to dynamically adjust (general-form) or optimally size (stature-based) the mechanical components (e.g. springs and motors) on wearable assistive devices.

We found high values of 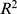 for linear curve fits to the moment-angle relationship at the ankle in both the dorsi-flexion and plantar-flexion phases (as shown in Table 1) that are in good agreement with previous results in the literature [34], [37]. However, we found that the ankle deviates from a linear behavior in the dual-flexion phase at a singular gait speed which is higher than the optimal gait speed; and the ankle behaves linearly at speeds above and below this speed. In fact, at slow gait speeds, the regression line of c-d in Fig. 1 has positive slope and the ankle experiences dorsi-flexion. As the gait speed increases towards the singular speed, the regression line becomes vertical and the ankle theoretically locks and exhibits infinite quasi-stiffness. For gait speeds faster than the singular speed, the regression line within interval c-d in Fig. 1 has negative slope and the ankle experiences plantar-flexion. Therefore, the ankle demonstrates dual behavior in this phase; mainly, dorsi-flexion at slow gaits and plantar-flexion at fast gaits. We further investigated possible correlation between this transfer speed and the body parameters 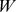 and 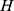; however, we did not find any notable correlation. Therefore, we speculate that this singular speed is primarily a preference of the subject; rather than governed by kinematic or kinetic constraints.

We observed that 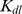 is highly dependent on 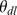. As a result, despite a relatively accurate prediction by the general-form models (22% error), the stature-based model that employs the average value of 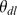 does not show promising predictions (error of 29%) for 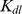 at the preferred gait speed. From a design point of view, since the range of values for 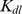 is substantially high and 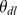 low, an assistive device for the ankle could employ some switching elements (e.g. a clutch) to lock the ankle in the dual-flexion phase, exhibiting infinite stiffness (e.g. similar to [67]).

Recently, researchers in the field of prosthetics and orthotics have moved toward quasi-passive systems and implemented impedance control methods in their designs [10], [12], [44], [47]. Previous research shows that *a priori* knowledge of the ankle quasi-stiffness variability is necessary, especially for the cases where the kinematic and kinetic data for a target subject are not known, which is usually the case in most design and fitting centers [13], [67], [68]. To develop prosthetic and orthotic devices, the designers obtain the gait lab kinematic and kinetic data of a sample healthy population and employ the *average* quasi-stiffness and work of the ankle joint to tune the prosthetic or orthotic device [13], [67], [68]. The average values range from 201 *Nm/rad* to 685 *Nm/rad* for 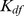, −655 *Nm/rad* to 229 *Nm/rad* for 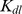, 201 *Nm/rad* for 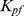, and ∼19.7 *J* for 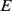, depending on the sample population that the designers have chosen [13], [67], [68]. The sample population is usually composed of individuals with weight, height, and preferred gait speed that are not necessarily representatives of the target user.

In order to examine the differences between a model based on average data and our models, we found the average quasi-stiffnesses and work for the gait data utilized in our study and examined the error between the quasi-stiffnesses and work predicted by the average and the true subject-specific quasi-stiffnesses and work. Table 4 compares the average error associated with the general-form models, stature-based models, and a model that merely uses the average values of 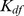, 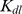, 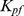, and 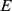 (as reported in Table 1). The results show much larger errors when the average values are utilized than with our models, suggesting that selection of the device stiffness and power based on the general-form models presented here may result in devices that more accurately mimic the gait of a healthy subject with similar gait and body parameters. Devices meant to operate mostly near the preferred gait speed could utilize a spring with stiffness equal to the quasi-stiffness of the ankle at the preferred gait speed (based on stature-based equations of Table 3). For other gait speeds, the stiffness of the device might ideally be dynamically ‘tuned’ based on the general-form equations presented in Table 2. For this purpose, the device would in a real-time mode measure the gait speed (e.g. using a GPS), ankle excursion (e.g. using a goniometer), and user’s weight and adapt the stiffness accordingly. All together, the models of this study may help researchers and clinicians tune the stiffness and power of orthotic and prosthetic devices according to the body size and gait speed of the user, and do so without needing to perform subject-specific gait analyses.

**Table 4:** Average Error Values for Different Models.

Parameter	General-Form	Stature-Based	Average Values
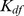	16%	20%	33%
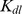	29%	29%	96%
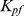	9%	13%	23%
*_E_*	25%	30%	82%

Applications of the models presented in this study are not restricted to the field of orthotics and prosthetics. These models could also be used for the design of ankle exoskeletons that are meant to augment the performance of healthy ankles. Researchers have proposed a range of sophistication in the design of exoskeletons from quasi-passive to fully active systems [3], [4], [6], [8]–[10]. The designers of active exoskeletons could utilize our equations for the propulsive work (Table 2 and 3) to size the active components (e.g. motors). Moreover, our findings suggest that the passive components (i.e. springs) could be further exploited in the design of these devices; provided that the passive components are properly tuned for the gait and user. For example, the design models of Table 3 suggest that the stiffness of an assistive device should ideally be chosen based on the weight and height of the subject.

Our study has a few limitations worth noting. First, we only addressed the behavior of the ankle during stance phase of normal walking on level ground. Our approach could be extended to other joints of the lower-limb, other gait regimes (e.g. running), and also account for variable terrain or carried loads. For example, in our preliminary study we showed that the quasi-stiffness of the ankle significantly increases as the ground slope changes [37].

Another limitation was that in order to establish the current models, we used 216 gait trials for 26 adult subjects. Therefore, our analyses could be generalized only to the range of age, height, weight, gait speed and mobility that the subjects represent and as much as the statistical significance supports. Our approach could be extended to other lower extremity joints, gait regimes (e.g. running), subject types (e.g. children), and also could be used to account for variable terrain or carried loaded. For example, researchers have shown significant dependence of the ankle quasi-stiffness on both gender and age [34]. Additionally, the data sets we used were obtained at three different gait labs under similar conditions (healthy adults walking on level ground), but with slight differences in protocols (e.g. overground vs. treadmill based data collection). On the one hand, the diversity of the data set in terms of walking speeds, and stature of the subjects should broaden the applicability of the statistical models of Table 2 and 3. On the other hand, some parameters, including age were not very variable- potentially limiting our confidence in predicting differences with respect to those factors. Finally, it is possible that methodological differences associated with the marker positioning for motion capture and the collection procedure for ground reaction force data (e.g. treadmill vs. overground) could have influenced the results. For example, the assumption that trials were all collected at constant gait speed is tougher to enforce using overground methods.

Finally, we employed several simplification and estimation steps to identify the important predictors that only hold when the subject walks in the sagittal plane with no pathologies in the gait. A more sophisticated model could relax these assumptions and take the eliminated terms and confined parameters into account. Moreover, the current work investigates the ankle behavior at a joint-level and does not consider the crosstalk between the adjacent joints. Future research should investigate the effect of interaction between the lower extremity joints caused by the function of biarticular muscles.

Taken together, we have established a family of models with different levels of sophistication that can predict the quasi-stiffness and propulsive work of the ankle in stance with relatively high accuracy. From an applied standpoint, our models might be used in gait analysis, modeling, and simulations, and also as a useful design tool in the fields of orthotics, prosthetics, and bipedal robots.

## Supporting Information

Figure S1
**A schematic model of the support foot for a subject walking in the sagittal plane.** The figure depicts the proximal and distal forces and moments applied on the foot, and the center of mass of the foot (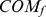). The ground reaction force and moment are also shown at the center of pressure (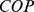).(TIF)

Table S1 **Description of mathematical expressions.**
(DOCX)

Appendix S1 **Inverse dynamics analysis.**
(DOCX)
