# Supplementary figures and images for: Estimation of Quasi-Stiffness and Propulsive Work of the Human Ankle in the Stance Phase of Walking
Source: PLoS One. 2013 Mar 21;8(3):e59935. doi: 10.1371/journal.pone.0059935 (PMC3605342; doi:10.1371/journal.pone.0059935)

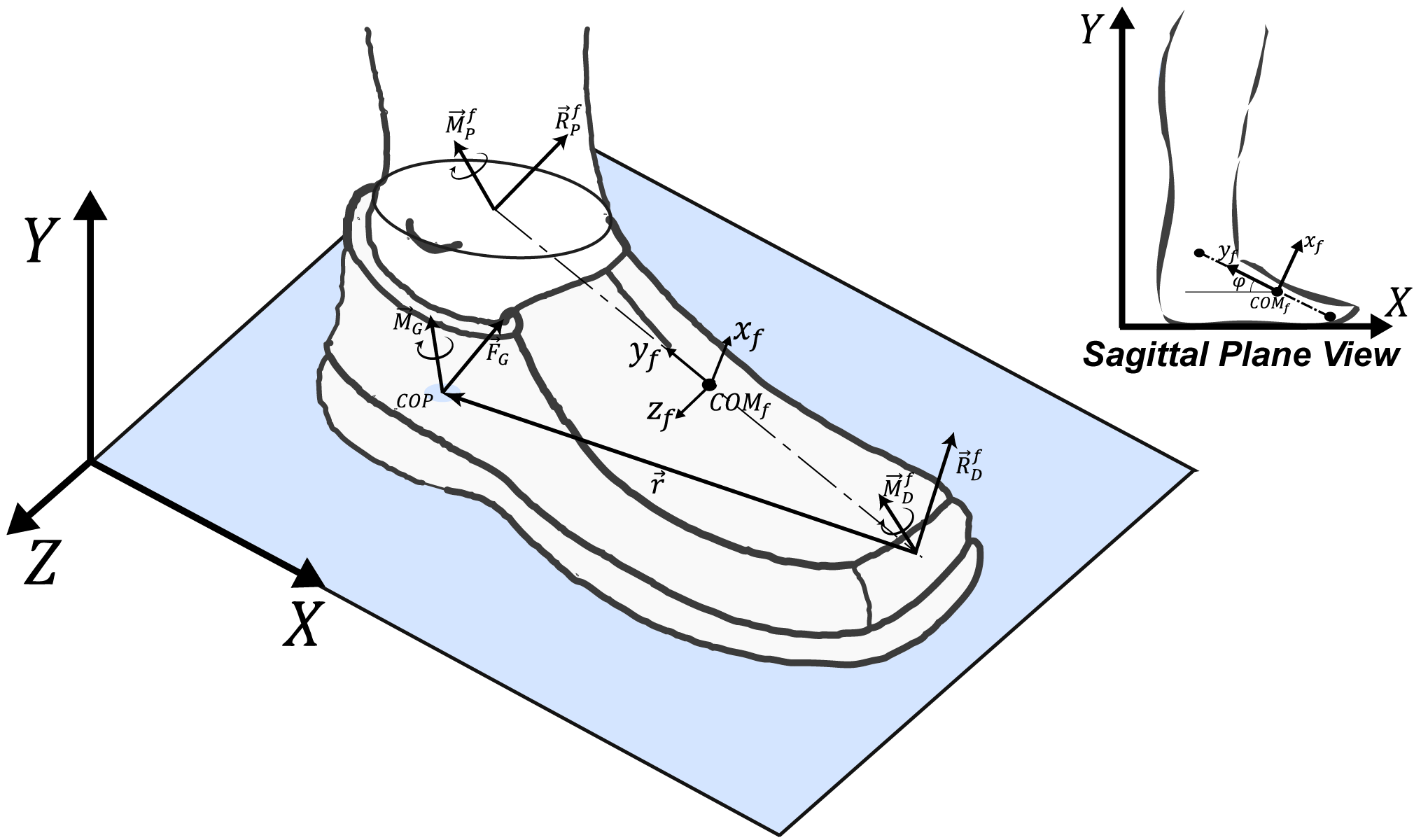

Supplement: Figure S1 — A schematic model of the support foot for a subject walking in the sagittal plane. The figure depicts the proximal and distal forces and moments applied on the foot, and the center of mass of the foot (). The ground reaction force and moment are also shown at the center of pressure (). (TIF) [file pone.0059935.s001.tif]
