# Supplementary material for: Estimation of Quasi-Stiffness and Propulsive Work of the Human Ankle in the Stance Phase of Walking
Source: PLoS One. 2013 Mar 21;8(3):e59935. doi: 10.1371/journal.pone.0059935 (PMC3605342; doi:10.1371/journal.pone.0059935)
Supplement: Table S1 — Description of mathematical expressions. (DOCX) [file pone.0059935.s002.docx]

**TABLE S1.** Description of the Mathematical Expressions

| **Parameter** | **Description** | **Parameter** | **Description** |
| --- | --- | --- | --- |
| $\boldsymbol{K}_{\boldsymbol{df}}$ | Ankle quasi-stiffness in dorsi-flexion phase of stance | ${\vec{\dot{\boldsymbol{\omega}}}}_{\boldsymbol{f}}$ | Angular acceleration of foot |
| $\boldsymbol{K}_{\boldsymbol{dl}}$ | Ankle quasi-stiffness in dual-flexion phase of stance | $\boldsymbol{COM}_{\boldsymbol{f}}$ | Center of mass of foot |
| $\boldsymbol{K}_{\boldsymbol{pf}}$ | Ankle quasi-stiffness in plantar-flexion phase of stance | ${\bar{\boldsymbol{e}}}_{\boldsymbol{Y}}$ | Unit vector vertical to the ground and along $Y$ |
| $\boldsymbol{\theta}_{\boldsymbol{df}}$ | Ankle excursion in dorsi-flexion phase of stance | ${\vec{\boldsymbol{U}}}_{\boldsymbol{f}}$ | Angular momentum of foot |
| $\boldsymbol{\theta}_{\boldsymbol{dl}}$ | Ankle excursion in dual-flexion phase of stance | $\left[ \boldsymbol{I}_{\boldsymbol{f}} \right]$ | Matrix of moment of inertia of foot |
| $\boldsymbol{\theta}_{\boldsymbol{pf}}$ | Ankle excursion in plantar-flexion phase of stance | ${\vec{\boldsymbol{M}}}_{\boldsymbol{P}}^{\boldsymbol{f}}$ | Foot proximal moment in global coordinate system |
| $\boldsymbol{W}$ | Body weight | ${\vec{\boldsymbol{M}}}_{\boldsymbol{D}}^{\boldsymbol{f}}$ | Foot distal moment in global coordinate system |
| $\boldsymbol{V}$ | Gait speed | ${\vec{\boldsymbol{M}}}_{\boldsymbol{p}}^{\boldsymbol{f}}$ | Foot proximal moment in foot anatomical coordinate system |
| $\boldsymbol{H}$ | Body height | ${\vec{\boldsymbol{M}}}_{\boldsymbol{d}}^{\boldsymbol{f}}$ | Foot distal moment in foot anatomical coordinate system |
| ${\vec{\boldsymbol{M}}}_{\boldsymbol{G}}$ | Ground reaction moment | ${\vec{\boldsymbol{R}}}_{\boldsymbol{P}}^{\boldsymbol{f}}$ | Foot proximal force in global coordinate system |
| ${\vec{\boldsymbol{F}}}_{\boldsymbol{G}}$ | Ground reaction force | ${\vec{\boldsymbol{R}}}_{\boldsymbol{D}}^{\boldsymbol{f}}$ | Foot distal force in global coordinate system |
| $\vec{\boldsymbol{r}}$ | Vector from toe to center of pressure | ${\vec{\boldsymbol{R}}}_{\boldsymbol{p}}^{\boldsymbol{f}}$ | Foot proximal force in foot anatomical coordinate system |
| $\boldsymbol{L}_{\boldsymbol{f}}$ | Length of foot | ${\vec{\boldsymbol{R}}}_{\boldsymbol{d}}^{\boldsymbol{f}}$ | Foot distal force in foot anatomical coordinate system |
| ${\bar{\boldsymbol{e}}}_{\boldsymbol{Y}}^{\boldsymbol{f}}$ | Unit vector along foot segment | $\boldsymbol{X-Y-Z}$ | Global coordinate system |
| $\boldsymbol{m}_{\boldsymbol{f}}$ | Foot mass | $\boldsymbol{x}_{\boldsymbol{f}}\boldsymbol{-}\boldsymbol{y}_{\boldsymbol{f}}\boldsymbol{-}\boldsymbol{z}_{\boldsymbol{f}}$ | Anatomical coordinate system of foot |
| ${\vec{\boldsymbol{a}}}_{\boldsymbol{f}}$ | Foot acceleration | $\boldsymbol{M}_{\boldsymbol{A}}^{\boldsymbol{Z}}$ | Ankle moment on the sagittal plane |
| $\boldsymbol{g}$ | Acceleration due to gravity | ${\vec{\boldsymbol{d}}}_{\boldsymbol{f}}$ | Vector connecting center of mass of foot to toe |
| $\boldsymbol{L}_{\boldsymbol{p}}^{\boldsymbol{f}}$ | Distance between center of mass of foot to ankle | ${\vec{\boldsymbol{p}}}_{\boldsymbol{f}}$ | Vector connecting center of mass of foot to ankle |
| $\left[ \boldsymbol{AG} \right]_{\boldsymbol{f}}$ | Transformation matrix from anatomical system of foot to global system | ${\vec{\boldsymbol{F}}}_{\boldsymbol{f}}$ | Any force applied on foot |
| $\left[ \boldsymbol{GA} \right]_{\boldsymbol{f}}$ | Transformation matrix from global system to anatomical system of foot | ${\vec{\boldsymbol{M}}}_{\boldsymbol{f}}$ | Any moment applied on foot |
| ${\vec{\boldsymbol{\omega}}}_{\boldsymbol{f}}$ | Angular velocity of foot | $\boldsymbol{Fr}$ | Froude number for walking |
