# Supplementary material for: Estimation of Quasi-Stiffness and Propulsive Work of the Human Ankle in the Stance Phase of Walking
Source: PLoS One. 2013 Mar 21;8(3):e59935. doi: 10.1371/journal.pone.0059935 (PMC3605342; doi:10.1371/journal.pone.0059935)
Supplement: Appendix S1 — Inverse dynamics analysis. (DOCX) [file pone.0059935.s003.docx]

# **APPENDIX: INVERSE DYNAMICS ANALYSIS**

In this section, we derive the equations of the reaction forces and moments for the ankle joint. We are mainly interested in a generic expression for the ankle moment. Winter presents a detailed explanation of the inverse dynamics analysis [52]. Our analyses develop the Newtonian equations of motion of foot with respect to (w.r.t.) the global coordinate system as shown by $X-Y-Z$ in Fig. S1. Next, using the Euler equations of motion, we derive an expression for the moment of the ankle joint w.r.t. the anatomical axes of the foot shown by $x_{f}-y_{f}-z_{f}$ in Fig. S1. The anatomical coordinate frame of the foot is established by placing $y_{f}$ on the axis connecting the toe to the ankle center, $z_{f}$ along with $Z$ and $x_{f}$ along with the cross-product of $y_{f}$ and $z_{f}$. The expression for the moment of ankle is transformed to $X-Y-Z$ to obtain an equation for the ankle moment in the global coordinate system. Table S1 lists the parameters that are used in this text and brief descriptions for each parameter.

The pair of ground reaction force ($\vec{F}_{G}$) and ground reaction moment ($\vec{M}_{G}$) is transferred to the distal joint (i.e. toe) for the center of pressure (COP) to obtain the distal force ($\vec{R}_{D}^{f}$) and the distal moment ($\vec{M}_{D}^{f}$) of the foot w.r.t. $X-Y-Z$. Hence w.r.t. $X-Y-Z$, we get:

$\vec{R}_{D}^{f}=\vec{F}_{G}$ (A-1-a)

$\vec{M}_{D}^{f}=\vec{M}_{G}-\vec{F}_{G}\times\vec{r}$ (A-1-b)

and w.r.t. $x_{f}-y_{f}-z_{f}$ we get:

$\vec{R}_{d}^{f}=\left[ GA \right]_{f}\vec{F}_{G}$ (A-2-a)

$\vec{M}_{d}^{f}=\left[ GA \right]_{f}\left( \vec{M}_{G}-\vec{F}_{G}\times\vec{r} \right)$ (A-2-b)

$\vec{r}$ is the vector connecting the distal joint to COP and $\left[ GA \right]_{f}$ is a proper (i.e. reserves inner product and has a determinant of 1) rotation from $X-Y-Z$ to $x_{f}-y_{f}-z_{f}$. The reaction force at the ankle ($\vec{R}_{P}^{f}$) is derived using the Newtonian equation of motion for the foot:

$\sum\vec{F}_{f}=m_{f}\vec{a}_{f}{\therefore\vec{R}}_{P}^{f}=\vec{R}_{D}^{f}+m_{f}\vec{a}_{f}+m_{f}g\bar{e}_{Y}$ (A-3)

where, $\vec{F}_{f}$ denotes any force that is applied on the foot segment and $\vec{a}_{f}$ is the acceleration of the center of mass of the foot (${COM}_{f}$). $m_{f}$ is the mass of the foot segment, $\bar{e}_{Y}$ is a unit vector along the $Y$-axis, and $g$ is the acceleration due to gravity. Equation (A-1-a) gives us the proximal force of the foot w.r.t. $X-Y-Z$ as:

$\vec{R}_{P}^{f}=\vec{F}_{G}+m_{f}\vec{a}_{f}+m_{f}g\bar{e}_{Y}$ (A-4-a)

which could be transformed to $x_{f}-y_{f}-z_{f}$ through a rotation as:

$\vec{R}_{p}^{f}=\left[ GA \right]_{f}\vec{R}_{P}^{f}$ (A-5)

Now, we develop the Euler equation of motion for the foot segment w.r.t. $x_{f}-y_{f}-z_{f}$ to derive an expression of the moment at its proximal joint (i.e. ankle):

$\sum\vec{M}_{f}=\left[ I_{f} \right]{\vec{\dot{\omega}}}_{f}+\vec{\omega}_{f}\times\vec{U}_{f}$ (A-6)

wherein, $\vec{M}_{f}$ denotes any moment that is applied on the foot segment and $\left[ I_{f} \right]$ is the matrix of moment of inertia. $\vec{\omega}_{f}$ the angular velocity, ${\vec{\dot{\omega}}}_{f}$ the angular acceleration, and $\vec{U}_{f}$ is the angular momentum of the foot segment. Expanding the left hand side of the above equation gives us:

$\vec{M}_{p}^{f}=\vec{M}_{d}^{f}-\vec{R}_{d}^{f}\times\vec{d}_{f}+\vec{R}_{p}^{f}\times\vec{p}_{f}+\left[ I_{f} \right]{\vec{\dot{\omega}}}_{f}+\vec{\omega}_{f}\times\vec{U}_{f}$ (A-7)

where,$\vec{M}_{p}^{f}$is the moment at the proximal joint of the foot segment expressed w.r.t. $x_{f}-y_{f}-z_{f}$. $\vec{d}_{f}$ is the vector that connects the center of mass of the foot (${COM}_{f}$) to the toe and $\vec{p}_{f}$ is the vector that connects ${COM}_{f}$ to the ankle joint both expressed w.r.t. $x_{f}-y_{f}-z_{f}$ . Here, the tip of the toe is chosen such that ${COM}_{f}$ relies on the origin of $x_{f}-y_{f}-z_{f}$. Now, we insert the corresponding terms in equation (A-7):

$\vec{M}_{p}^{f}=\left[ GA \right]_{f}\left( \vec{M}_{G}-\vec{F}_{G}\times\vec{r} \right)-\left[ GA \right]_{f}\vec{F}_{G}\times\vec{d}_{f}+\left[ GA \right]_{f}\left( \vec{F}_{G}+m_{f}\vec{a}_{f}+m_{f}g\bar{e}_{Y} \right)\times\vec{p}_{f}+\left[ I_{f} \right]{\vec{\dot{\omega}}}_{f}+\vec{\omega}_{f}\times\vec{U}_{f}$ (A-8)

Since $\left[ GA \right]_{f}$ is a proper rotation, $\vec{M}_{p}^{f}$ could be transformed into $X-Y-Z$ as:

$$\vec{M}_{P}^{f}=\left( \vec{M}_{G}-\vec{F}_{G}\times\vec{r} \right)-\vec{F}_{G}\times\left[ AG \right]_{f}\vec{d}_{f}+\left( \vec{F}_{G}+m_{f}\vec{a}_{f}+m_{f}g\bar{e}_{Y} \right)\times\left[ AG \right]_{f}\vec{p}_{f}$$

$+\left[ AG \right]_{f}\left( \left[ I_{f} \right]{\vec{\dot{\omega}}}_{f}+\vec{\omega}_{f}\times\vec{U}_{f} \right)$ (A-9)

where, $\left[ AG \right]_{f}=\left[ GA \right]_{f}^{-1}$. One should notice that:

$\vec{F}_{G}\times L_{f}\bar{e}_{Y}^{f}= -\vec{F}_{G}\times\left[ AG \right]_{f}\vec{d}_{f}+\vec{F}_{G}\times\left[ AG \right]_{f}\vec{p}_{f}$ (A-10)

where, $L_{f}$ is the length of the foot segment and $\bar{e}_{Y}^{f}$ is the unit vector along the $y_{f}$-axis of the foot segment expressed w.r.t. $Y$-axis. Thus, equation (A-9) can be written in the following form:

$\vec{M}_{P}^{f}=\left( \vec{M}_{G}-\vec{F}_{G}\times\vec{r}+\vec{F}_{G}\times L_{f}\bar{e}_{Y}^{f} \right)+\left( m_{f}\vec{a}_{f}+m_{f}g\bar{e}_{Y} \right)\times L_{p}^{f}\bar{e}_{Y}^{f}+\left[ AG \right]_{f}\left( \left[ I_{f} \right]{\vec{\dot{\omega}}}_{f}+\vec{\omega}_{f}\times\vec{U}_{f} \right)$ (A-11)

where, $L_{p}^{f}$ is the distance between ${COM}_{f}$ and the ankle.
